# Growth modulation and metabolic responses of *Ganoderma boninense* to salicylic acid stress

**DOI:** 10.1371/journal.pone.0262029

**Published:** 2021-12-31

**Authors:** Cu Ean Ong, Rafidah Ahmad, You Keng Goh, Kamalrul Azlan Azizan, Syarul Nataqain Baharum, Kah Joo Goh

**Affiliations:** 1 Advanced Agriecological Research Sdn. Bhd., Kota Damansara, Petaling Jaya, Selangor, Malaysia; 2 Metabolomics Research Laboratory, Institute of Systems Biology (INBIOSIS), Universiti Kebangsaan Malaysia (UKM), Bangi, Selangor, Malaysia; Fujian Agriculture and Forestry University, CHINA

## Abstract

Various phenolic compounds have been screened against *Ganoderma boninense*, the fungal pathogen causing basal stem rot in oil palms. In this study, we focused on the effects of salicylic acid (SA) on the growth of three *G*. *boninense* isolates with different levels of aggressiveness. In addition, study on untargeted metabolite profiling was conducted to investigate the metabolomic responses of *G*. *boninense* towards salicylic acid. The inhibitory effects of salicylic acid were both concentration- (*P < 0*.*001*) and isolate-dependent (*P < 0*.*001*). Also, growth-promoting effect was observed in one of the isolates at low concentrations of salicylic acid where it could have been utilized by *G*. *boninense* as a source of carbon and energy. Besides, adaptation towards salicylic acid treatment was evident in this study for all isolates, particularly at high concentrations. In other words, inhibitory effect of salicylic acid treatment on the fungal growth declined over time. In terms of metabolomics response to salicylic acid treatment, *G*. *boninense* produced several metabolites such as coumarin and azatyrosine, which suggests that salicylic acid modulates the developmental switch in *G*. *boninense* towards the defense mode for its survival. Furthermore, the liquid chromatography time-of-flight mass spectrometry (LC-TOF-MS) analysis showed that the growth of *G*. *boninense* on potato dextrose agar involved at least four metabolic pathways: amino acid metabolism, lipid pathway, tryptophan pathway and phenylalanine pathway. Overall, there were 17 metabolites that contributed to treatment separation, each with *P<0*.*005*. The release of several antimicrobial metabolites such as eudistomin I may enhance *G*. *boninense*’s competitiveness against other microorganisms during colonisation. Our findings demonstrated the metabolic versatility of *G*. *boninense* towards changes in carbon sources and stress factors. *G*. *boninense* was shown to be capable of responding to salicylic acid treatment by switching its developmental stage.

## 1. Introduction

*Ganoderma boninense* Pat., the causal agent of basal and upper stem rot of oil palm (*Elaeis guineensis*), is a major threat to the oil palm industry in Southeast Asia [1]. *Ganoderma boninense* is a hemibiotrophic fungus, where it first colonizes both root cortex and stem bases intracellularly [2]. The latter necrotrophic phase involves extensive degradation of host cell wall [2]. This fungus degrades palm lignin to access the energy-rich cellulose by secreting an array of ligninolytic enzymes [3, 4] and causes chlorotic lesion in the bole [5]. Due to the progressive degradation of root system and subsequently the lower stem, translocation of water and nutrients will be affected [2, 6]. Therefore, infected palms are often associated with moisture stress symptoms, i.e., multiple unopened spears, pale and progressively smaller canopy [5, 7]. It is unfortunate that there is no effective control measure for this disease, although numerous methods have been tried [8]. These include the application of phenolic compounds, which is widely tested against various pathogens and host plants due to its dual roles in enhancing plant defense and inhibiting growth and development of pathogens.

Supplementation of phenolic compounds were proposed as a method to promote oil palm tolerance towards *G*. *boninense* [9–12]. For instance, salicylic acid (SA) is a key plant phenolic that acts as a signaling molecule to activate local and systemic acquired resistance (SAR) against biotrophic pathogens. Consequently, the plant will express pathogenesis-related proteins having antimicrobial activity and/or able to confer resistance against the pathogens [13]. The inhibitory effect of SA has been proven against several bacteria and fungi, for example in *Penicillium expansum*, *Sclerotia rolfsii*, *S*. *minor*, and *Pectobacterium caratovorum*, to name a few. Salicylic acid significantly reduced mycelial growth and conidial germination of *P*. *expansum* [14]. As for *S*. *rolfsii* and *S*. *minor*, SA treatment inhibited both fungal growth and sclerotial differentiation [15]. Complete inhibition of the growth of *P*. *caratovorum* was observed at high SA concentrations, whereas low SA concentrations reduced the bacterial count [16].

SA undoubtedly plays a pivotal part in plant defense against plant pathogens. However, as reviewed recently by Qi et al.(2018) [17], plant pathogens have developed several strategies in overcoming SA to ensure successful colonisation and infection of plant hosts. These strategies include reduction of SA accumulation, disruption of SA biosynthesis and interference in SA downstream signaling [17]. The inhibitory effect of SA on the growth of *G*. *boninense* has been reported [11, 12]. However, to the best of our knowledge, there is no information on the defense mechanism of *G*. *boninense* towards SA treatment, especially with respect to its metabolic response.

Defense mechanism of *G*. *boninense* towards SA, which is a stress factor induced during pathogen invasion, is an important piece of puzzle for the understanding of host-pathogen interactions. Rees et al. (2009) [2] postulated the role of melanized pseudo-sclerotia in protecting *G*. *boninense* against host defenses. This structure is commonly formed at the third phase of infection, during necrotrophic stage where host cell wall is heavily degraded by the activity of ligninolytic enzymes [2, 18]. Additionally, *G*. *boninense* may degrade SA to mitigate the treatment effect, as reported by Chong (2010) [9] whereby the fungus degrades other phenolic compounds into less- or non-antifungal compounds.

Thus, our primary objective was to investigate the metabolomics responses of *G*. *boninense* when challenged with SA through untargeted metabolite profiling analysis. In addition, the inhibitory effects of SA on the growth of three *G*. *boninense* isolates with different pathogenicity levels were also investigated.

## 2. Materials and methods

### 2.1 Fungal cultures and growth conditions

Three *G*. *boninense* isolates were tested in this study, i.e., G8 (least aggressive), PER71 (moderately aggressive), and G10 (most aggressive), each with different levels of aggressiveness in causing disease in the nursery screening [19]. These isolates were identified to be *G*. *boninense* via molecular characterization [19]. The cultures were grown on malt extract agar (MEA) (Difco, Becton Dickinson Diagnostics, Sparks, Maryland) at 24°C in the dark for 14 days prior to the commencement of experiment.

### 2.2 Poison medium assay

Poison medium assay was conducted according to Bivi et al. (2012) [11] with minor modifications. In our experiment, salicylic acid (SA) powder (Sigma-Aldrich, St. Louis, Missouri) was first dissolved in absolute ethanol (approximately 3mL) due to its poor solubility in distilled water. Distilled water was then added into the dissolved SA to prepare the working solution. SA solution was filter-sterilized before adding into potato dextrose agar (PDA) (Difco, Becton Dickinson Diagnostics, Sparks, Maryland). The final concentrations of SA in PDA were 50, 100, 150 and 200 μg g^-1^. For positive control, SA was replaced by distilled water with minimal amount of absolute ethanol (0.24% v/v). There was one positive control for each isolate of *G*. *boninense*. Ten millimeter of *G*. *boninense* mycelial plugs from the culture plates were transferred to SA-amended and positive control petri dishes with a diameter of 88 mm. There were five replicates for each treatment and control. The cultures were kept under the same growing conditions as culture maintenance on MEA.

Radial growth of the fungal mycelia was measured and recorded on days 5, 7, 9 and 12, after inoculation. These measurements were used to calculate percent inhibition of radial growth (PIRG) as described by Skidmore and Dickinson (1976) [20]. The percent difference in radial growth between the positive control and treated culture was used to determine the treatment effect on the fungal growth as shown below:

$$PIRG\;(\%)=\frac{Mycelia\;growth\;in\;control-mycelia\;growth\;in\;treatment\;}{Mycelia\;growth\;in\;control}X\;100\%$$

### 2.3 Metabolite extraction

For metabolite profiling, there were four treatments in 2^2^ factorial combinations: non-inoculated plate without SA (C0), non-inoculated plate with 200 μg g^-1^ SA (C200), *Ganoderma* G10-inoculated plate without SA (G0), and *Ganoderma* G10-inoculated plate with 200 μg g^-1^ SA (G200). There were three biological replicates with three technical replicates for each treatment. In total, there were 36 samples for the metabolomics analysis.

Metabolite extraction for untargeted metabolomics analysis was optimized by Lim et al. (2018) [21], specifically for investigating the metabolomics responses of *G*. *boninense* to external stimuli *in vitro*. We had adapted their method with minor modifications in our study. Briefly, two culture media with the size of 1.5 x 3.0 cm, and approximately 1.0 cm from the edge of fungal mycelial plug, were obtained for metabolite extraction. Cold methanol at 60% concentration was added to the samples at a ratio of 1g mL^-1^ (1:1, sample mass: methanol ratio) and vortexed vigorously. The samples were then frozen in liquid nitrogen for 5 minutes and allowed to thaw on ice for 10 minutes. A sonic dismembrator (Fisher Scientific, FB120) fitted with a Model CL-18 probe was used to homogenize the sample. Sonication was carried out at 65% power and 30% amplitude to provide 15 second pulses. The sonication step was repeated five times for each sample with 1 minute break between each repetition. The homogenized samples were then centrifuged at 13,000 rpm for 10 minutes at 4°C. After the centrifugation, supernatant was collected and transferred to a new cold tube. Precipitated pellet was re-extracted with 60% cold methanol, following the same procedure as described above. Supernatant from the first and second extractions were pooled into one sample and concentrated for at least 4 hours using vacuum concentrator (Eppendorf Concentrator Plus, Germany). The samples were then stored at -80°C prior to liquid chromatography-time-of-flight-mass spectrometry (LC-TOF-MS) analysis.

### 2.4 LC-TOF-MS analysis

The analytical platform used in this study was ultrahigh-performance liquid chromatography (UHPLC), Ultimate 3000 UHPLC System (Dionex, Sunnyvale,USA) connected to a time-of-flight mass spectrometry. Briefly, the extractions (1μl) were injected onto C18, reversed-phase column, AcclaimTM Polar Advantage II, 3 x 150 mm, 3 μm particle size in the UHPLC system using MiliQ water and 0.1% formic acid (A) and 100% acetonitrile (B) as mobile phase with total runtime of 22 minutes. The elution gradient was programmed as follows: a) initially the elution was isocratic at 5% B for 3 mins, b) 5% to 80% B (3–10 min), c) again, the elution was isocratic at 80% B (10–15 min) d) the column was re-equilibration at 5% B for 7 min before injecting the next sample. A constant flowrate at 0.4 mL min^-1^ and column temperature of 40°C were applied throughout the analysis. The mass spectrometry condition was performed on a microTOF Q III mass spectrometer (Bruker Daltonics, Bremen, Germany) equipped with an electrospray ionization interface (ESI), operating in positive mode. The parameters of the ESI source was set as follows: ion spray voltage, 4500 V at 200°C; nebulizer pressure, 1.2 bar; drying gas, 8 L min^-1^. The mass spectra of each compound were acquired over a mass range from 100 to 1000 m/z. The raw data obtained was processed using software, Data Analysis 4.0 and Profile Analysis (Bruker Daltonics) [22].

### 2.5 Data processing and data analysis

All mass spectral data were acquired using Data Analysis software (version 4.0, Bruker Daltonics). Raw data (.d) files were imported into profile analysis software (Bruker Daltonics) for further data processing, including peak alignment and peak normalization.

To determine the response of *G*.*boninense* treated with SA, we performed multivariate statistical analysis including principal component analysis (PCA) and partial least square-discriminant analysis (PLS-DA) using SIMCA-P+ software (version 12) (Umetrics, Umea, Sweden). All multivariate models were generated from the signal intensity data that was previously pre-processed using profile analysis software and has been applied with logarithmic transformation and auto-scaling (pareto) [23].

Other data analyses, such as heat map, was performed using MetaboAnalyst 5.0 software to visualize the metabolite profiles and reveal the relationship between metabolites and treatments. The analysis was performed by using the extracted dataset of 17 metabolites filtered by ANOVA, *p* < 0.005.

### 2.6 Metabolite identification

Retention time, mass-to-charge ratio (m/z), and fragmentation pattern were used to identify the metabolites, using ChemSpider, MassBank and KEGG database. Only limited information on metabolites was available for *G*. *boninense*, unlike *G*. *lucidum*. Hence, the functions of detected metabolites were frequently referred to their roles in *G*. *lucidum*. We also referred to other fungi and bacteria in deducing the roles of each metabolite.

### 2.7 Statistical analysis- ANOVA

Analysis of variance (ANOVA) was conducted using the PIRG values obtained from the poison medium assay using GenStat (18^th^ edition). The mean values obtained were compared using Least Significant Differences at 5% level. For metabolomics study, ANOVA and post-hoc Turkey’s test were used to validate the significance of the differential metabolites obtained from the multivariate data analysis.

## 3. Results

### 3.1 Effect of salicylic acid on the growth of *Ganoderma boninense*

**Fig 1:**
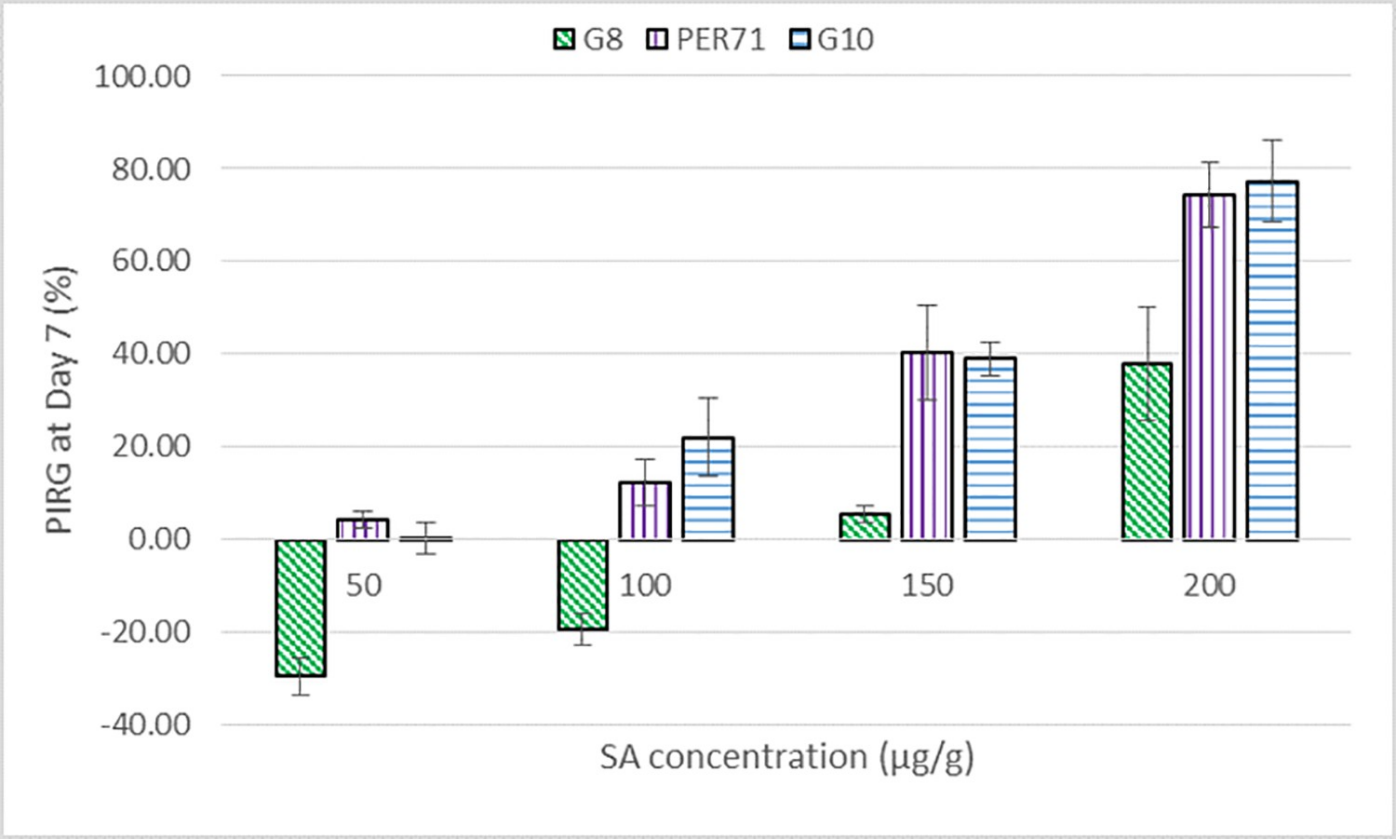
Average percent inhibition of radial growth (PIRG) of three *G*. *boninense* isolates, namely G8 (least aggressive), PER71 (moderately aggressive) and G10 (most aggressive) isolates, treated with different concentrations of salicylic acid, at day 7 after treatment, [n = 5].

**Fig 2:**
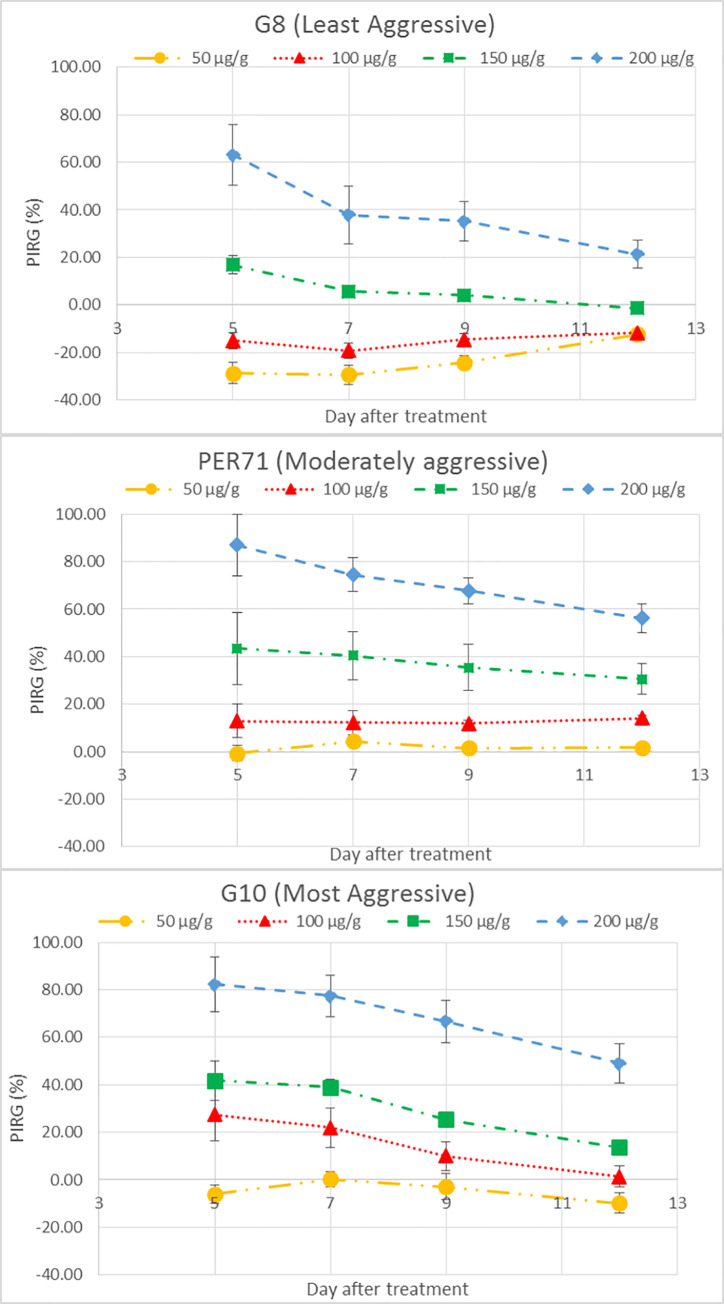
Average percent inhibition of radial growth (PIRG) of three *G*. *boninense* isolates treated with different concentrations of SA, at days 5, 7, 9, and 12 after treatments.

The inhibition of radial growth (%) as an effect of SA treatment are shown in Figs 1 and 2. The effect of SA on the growth of *G*. *boninense* was both concentration- (*P <0*.*001*) and isolate-dependent (*P <0*.*001*). The highest PIRG of 87% was obtained when PER71 isolate was treated with the highest concentration of SA (200 μg g^-1^), at day 5 after treatment (Fig 2). At low concentrations of 50 and 100 μg g^-1^, minimal inhibitory effect (≤ 20%) was observed for all three isolates (Figs 1 and 2). Noticeably, SA treatment had limited inhibitory effect on the least aggressive *G*. *boninense* G8 isolate up to 150 μg g^-1^ and at low concentrations of 50 and 100 μg g^-1^, it actually promoted the fungal growth (Fig 1 and S1 Fig).

In the presence of SA regardless of their concentrations, pale reddish zone formed around the fungal mycelia (S2–S4 Figs) of all *G*. *boninense* isolates cultured on the SA-amended-PDA medium. There was no color change in the other plates, i.e., in non-inoculated plate with (C200) and without SA (C0), and *Ganoderma*-inoculated plates without SA amendment (G0). In addition, SA treatment hastened the formation of melanized mycelia, and influenced the intensity of pigmentation (S2–S4 Figs). Both the formation of melanized mycelia and intensity of pigmentation were concentration-dependent.

### 3.2 Untargeted LC-TOF-MS

**Fig 3:**
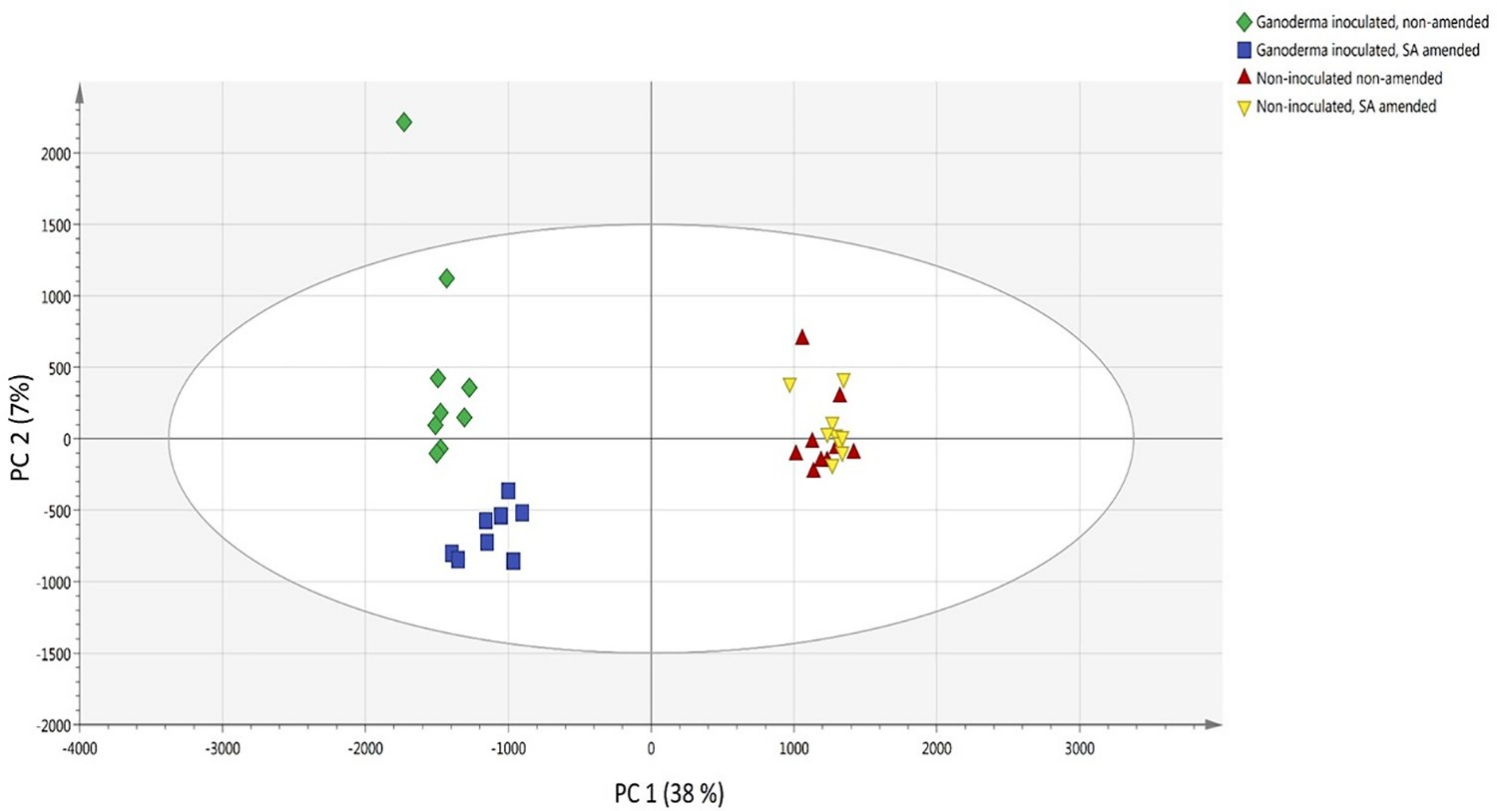
PCA score plot of PC1 versus PC2 scores for compounds or metabolites detected in each treatment: *Ganoderma*-inoculated (G), non- inoculated (C), with (200), and without salicylic acid (0) treatments.

After obtaining the LC-TOF-MS data matrices, PCA analysis (Fig 3) was conducted to evaluate the differences in the acquired LC-TOF-MS Base Peak Chromatograms to maximize the difference of metabolites between treatments. Out of the 36 samples, one sample (G200-R1A) did not passed internal QC requirements due to technical error and was excluded from the subsequent analysis. The first two principal components (PC1 and PC2) accounted for 38% and 7% of total variation, respectively, separated the control (non-inoculated plates) and the treatment groups (*Ganoderma*-inoculated plates) (Fig 3).

**Fig 4:**
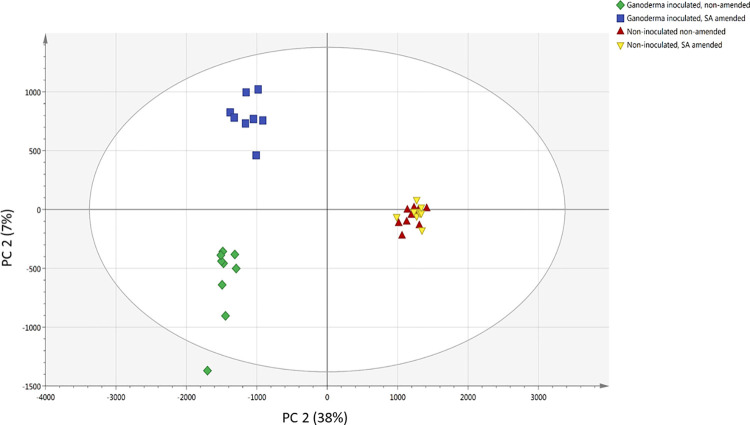
PLS-DA score plots for compounds or metabolites detected in each treatment: *Ganoderma*-inoculated (G), non- inoculated (C), with (200), and without salicylic acid (0) treatments. Each point on the scatter plot refers to a single sample, with R^2^X (cumulative) = 0.448, R^2^Y (cumulative) = 51.4% and Q^2^ (cumulative) = 0.541. The eclipse represent the 95% confidence interval.

PLS-DA model (Fig 4) was then applied to detect the most influential metabolites for discrimination between the different treatments. The predictive quality of the PLS-DA model for the first two components was good (Fig 4). The cumulative variation modelled in the X-matrix using three PLS factors was 44.8% (R^2^Xcum = 0.448) and 61.9% (R^2^Ycum = 0.619) in the Y matrix (Fig 4). The cross-validation parameter Q^2^cum, which describes the predictive ability of the model, was 54.1% (Q^2^cum = 0.541). The Q^2^ value in this study was above 0.4, the threshold acceptable value for a biological model [24].

Based on the PLS-DA model, control groups (both C0 and C200) were separated from the *Ganoderma*-inoculated groups (both G0 and G200) along the PC1 axis. At the same time, G0 group was separated from G200 group along the PC2 axis (Fig 4). One of the G0 samples (G0-R2B) was outside the 95% Hotelling’s T squared ellipse in both PCA and PLS-DA plots. Based on the T2 range analysis, this sample surpassed the T2critic (95%) limits (S5 Fig). However, DModX plot of the PCA data indicated that there were no samples that exceed the threshold for rejecting a sample. DModX-distance for an outlier should be at least twice the Dcrit value (critical value of DModX) [25, 26]. G0-R2B is at the borderline of the D-Crit limit (0.05) and hence, was not an outlier and was included in the model calculation (S6 Fig).

**Fig 5:**
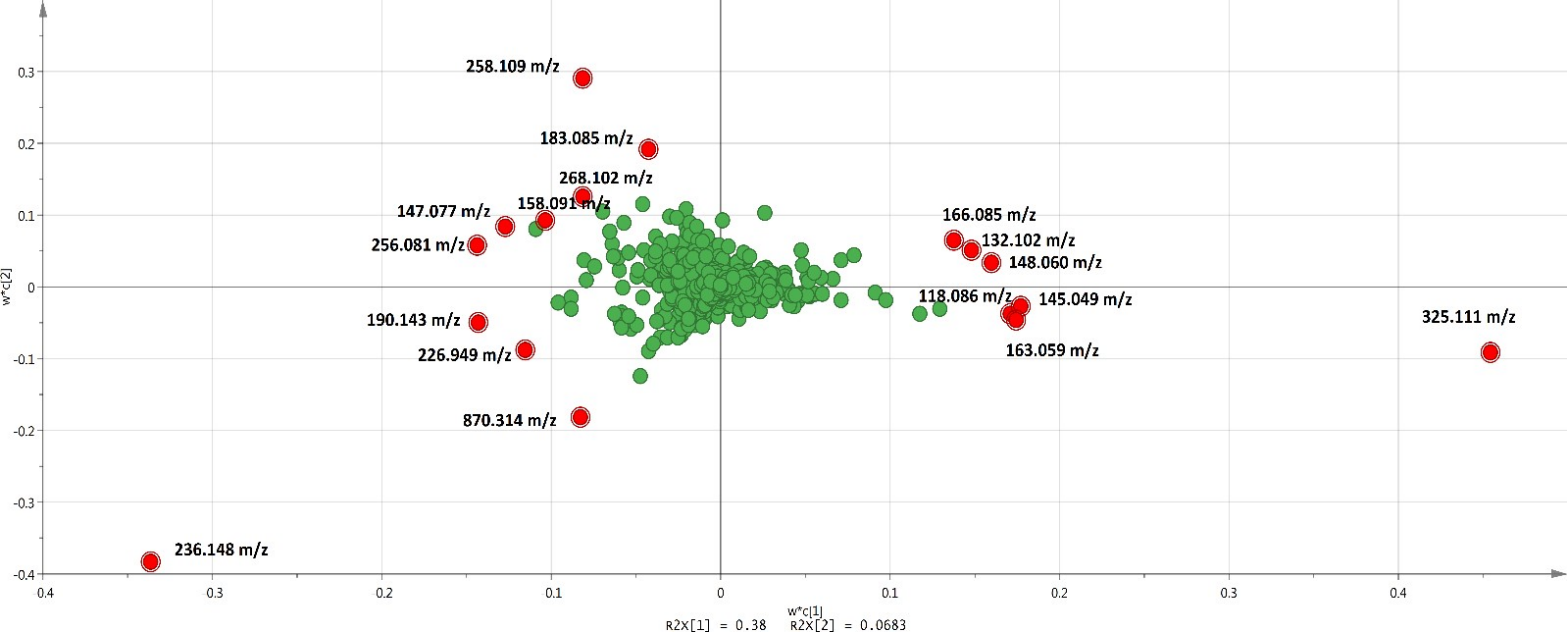
Loading plots of PLS-DA for metabolites (masses) detected via untargeted LC-TOF-MS, in different treatment. The green dot represents the masses distributed in w*c(1) and w*c(2) planes. The metabolites with the highest VIP (Variables Importance for the Projection) > 3 are highlighted in red dot.

All the variables were interpreted in loading plots (Fig 5). A total of 1154 metabolites was detected in the LC-MS analysis. The most important metabolites (mass) contributing to the apparent discrimination are listed in Table 1. All the metabolites in Table 1 that contributed to treatment separation were significant at *P<0*.*005*. As shown in Table 1 and Fig 5, amino acids and sugars, which are the constituents of potato dextrose agar, were detected in significantly higher amount in C0 and C200 treatments, and clearly defined the separation between non-inoculated (C groups) and *Ganoderma*-inoculated (G groups).

**Table 1:** List of metabolites with VIP value greater than 3 (in descending order) and their functions.

Retention time, Precursor ion m/z	VIP score	Fragmentation MS/MS	Adduct	Possible Identity	Classification	Function(s) from literature	Reference(s)
2.68 min, 236.148 m/z	12.1867	131.0692 5114 144.1008 3786 159.0605 1402 235.9798 3225 218.1372 1218	M+H^+^	Eudistomin I	Pyridine/ Carboline	β- carboline derivatives with anti-microbial activity. Eudistomin I was first isolated from marine tunicate species.	[27]
2.55 min, 325.111 m/z	11.4826	145.0501, 21974 127.0402, 21390 163.0604, 3859 109.0310, 3410 115.0403 1938 146.0539 1463 128.0434 1445	M+H^+^	Difructose anhydride I	Disaccharide	Smallest cyclic disaccharide consisting of two fructose units linked at their reducing carbons. Produced from degradation of inulin or levan.	[28]
2.45 min, 258.109 m/z	7.01702	112.0874 6626 113.0866 301 114.0971 189 115.0855 209 141.0644 1081 157.9836 161	M+H^+^	Coumarin, 3- (piperidinocarbonyl)-	Heterocylic compound	Phenylpropanoid compounds produced by plants upon abiotic and biotic stresses. May acts as antioxidant compound against reactive oxygen species (ROS).	[29]; [30]
2.60 min, 870.314 m/z	4.6896	204.0815, 34918 222.0912, 14809 366.1303, 7709 384.1429, 7052 168.0612, 4581 138.0522, 3273 205.0841, 3012 126.0520, 1971	M+H^+^	L-seryl- L- prolyl- L-threonyl- L-seryl-L-seryl- L-alanyl-L-seryl-L- seryl-L- phenylalanine (ChemSpider)	Aromatic amino acid	An important component in protein synthesis and a precursor for a wide range of secondary metabolites. Can be synthesized by fungi via shikimate pathway. Intermediate metabolite to produce coumarin.	[31]; [32]; [33]; [34]
2.54 min, 183.085 m/z	4.55184	110.0719 151 111.0461 134 129.0509 224 139.5482 104 156.0752 178 159.9671 188 181.9472 142	M+H^+^	Azatyrosine	Amino acid	Antibiotic and antitumor. Azatyrosine inhibits the chemical carcinogenesis involved in *ras* activation *in vivo* by preventing tumor formation. Tyrosine can be synthesized into DOPA-melanin via tyrosinase activity.	[35]; [36]; [37]; [38]
2.55 min, 163.059 m/z	4.46928	117.4101, 57 126.6273, 112 127.0458, 57 130.6880, 56 134.9737, 83 150.6416, 60 153.9557 63	M+H^+^	1,5- anhydro-D-fructose	Monosaccharide	Functional monosaccharide formed from starch and glycogen by α-1,4-glucan lyase (i.e., lytic degradation) Anti-cariogenic agent as it interfere the plaque-forming in *Streptococcus mutans*, ultimately inhibits the bacterial growth.	[39]; [40]
2.54 min, 145.049 m/z	4.45205	113.0238, 5652	M+H^+^	Dimethyl fumarate	Organic acid	Can be derived from fumaric acid which can be found in potato tubers.	[41]; [42]
2.51 min, 118.086 m/z	4.3345	117.0698, 138 87.0711, 83 57.1971, 90	M+H^+^	L-valine*	Amino acid	One of the essential amino acids in potato. One of the building blocks of enniatins i.e., phytotoxin produced by *Fusarium* which has antimicrobial activity against *Mycobacterium* spp. *Staphylococcus* spp., *E*. *coli*.	[43]; [44]
2.42 min, 148.060 m/z	4.03983	121.0263 71 130.0504 6090 130.7064 75 131.0501 981 131.9140 71 147.0508 94	M+H^+^	Glutamate*	Amino acid	One of the amino acids that can be found in potato.	[45]
3.77min, 132.102m/z	3.86381	51.8298 52 53.5151 45 113.9645 138 116.0699 83 131.9761 55	M+H^+^	L-leucine*	Amino acid	One of the essential amino acids in potato.	[43]
2.85 min, 256.081 m/z	3.81841	116.0697, 459 124.0370, 13061 125.0393, 1082 127.6498, 331 128.5129, 322 128.7825, 466 132.1941, 329 133.0463 608 135.3306 360 213.9177 684	M+H^+^	Indole-3-propanol phosphate	Anthranilate synthase component	Involved in the synthesis of L- tryptophan in bacteria.	[46]
6.40 min, 166.085 m/z	3.73964	120.0785,8500 121.0806,572 145.0443,305 163.1807,313	M+H^+^	D- phenylalanine*	Amino acid	Anti-bacterial against *Pseudoalteromonas* sp. by inhibiting biofilm formation.	[47]
2.14 min, 190.143 m/z	3.72969	120.0660 151 144.1401 4672 145.1418 737 146.1471 175 172.9564 161 189.2734 191 189.9833 276	M+H^+^	Nitric acid	Non-carboxylic acid/ Ester	Nitric acid is less discussed in fungal physiology compared to nitric oxide (NO). NO involved in fungal structural development, i.e. conidiation, appressorium maturation, sporulation. NO stimulates the formation of fruiting bodies in *Flammulina velutipes*. NO was also reported to influence programmed cell death (PCD) in higher eukaryotes. In addition, NO was able to activate various antioxidant genes in *E*. *coli* and *B*. *subtilis* against oxidative and nitrosative stress.	[48]
2.32 min, 147.077 m/z	3.70168	70.9368 111 94.1302 129 103.9512 105 116.9536 113 129.5265 72 130.0497 5620 131.0520 253 132.0567 168	M+H^+^	Glutamine	Amino acid	Involved in D-glutamine and D-glutamate metabolism.	[45]
5.40 min, 268.102 m/z	3.53934	136.0613 14139 165.0501 88 182.0791 157	M+ACN+Na	Tryptophan*	Amino acid	One of the essential amino acids in potato.	[43]
1.86 min, 226.949 m/z	3.52431	156.0779 174 158.9661 101 159.9049 114 159.9776 96 167.0950 96 181.0991 108 181.9471 444	M+H^+^	Chorismatic acid	Organic acid	Contributes skeleton for tryptophan synthesis.	[49]
2.86 min, 158.091 m/z	3.36871	112.0874 6626 113.0866 301 114.0971 189 115.0855 209 141.0644 1081 157.9836 161	M+H^+^	2-aminomuconate	Fatty acyl	An intermediate product in tryptophan degradation pathway to yield acetyl co-A.	[50]

*Compounds that were identified by authentic standard.

**Fig 6:**
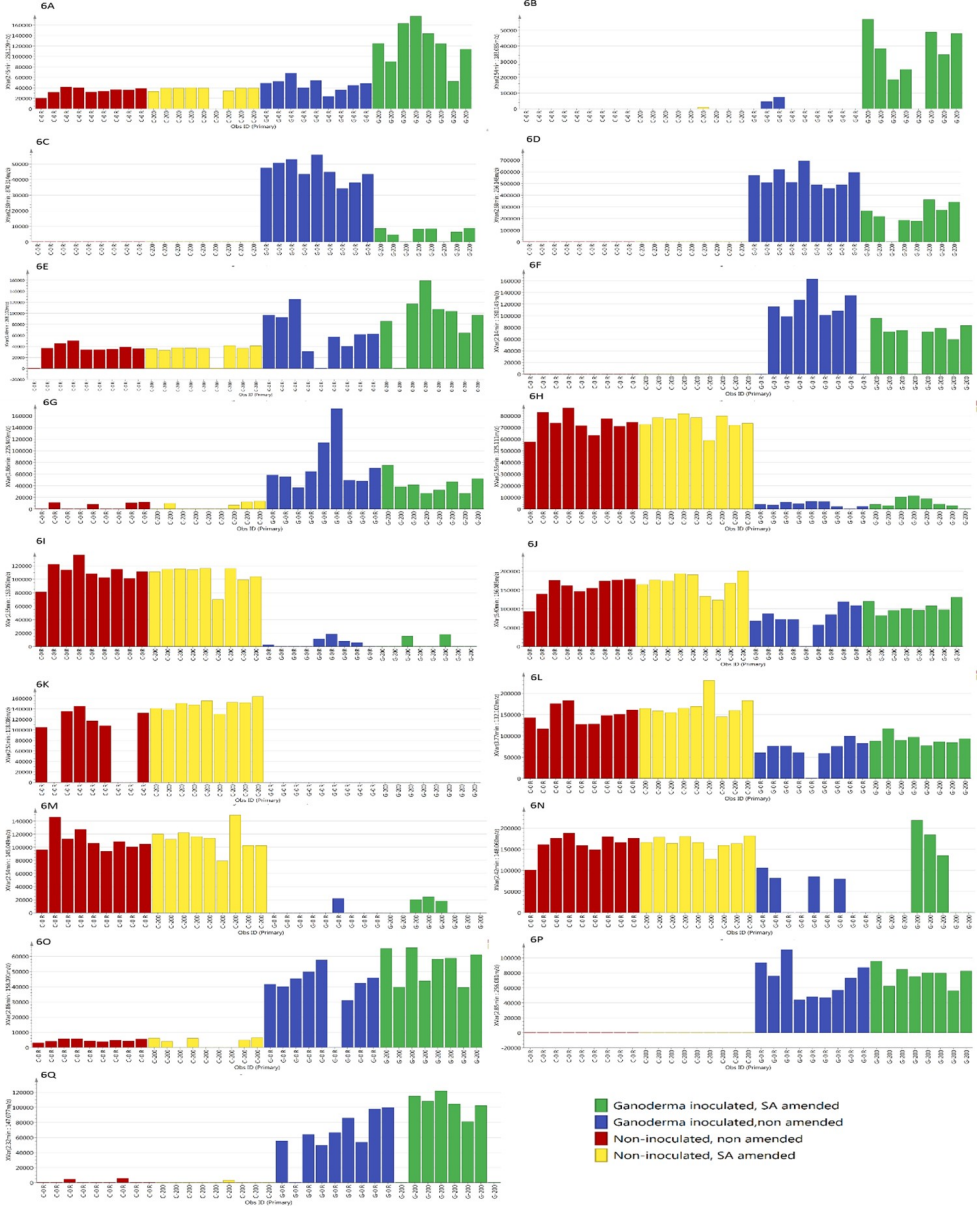
Representative ion intensity for the *m*/*z* value (**A**) coumarin, 3- (piperidinocarbonyl)- (258.109 m/z), (**B**) azatyrosine (183.085 m/z), (**C**) L-seryl-L-prolyl-L-threonyl- L-seryl-L-seryl- L-alanyl-L-seryl-L- seryl-L-phenylalanine (870.314 m/z), (**D**) eudistomin I (236.148 m/z), (**E**) tryptophan (268.102 m/z), (**F**) nitric acid (190.143 m/z), (**G**) chorismatic acid (226.949 m/z), (**H**) difructose anhydride (325.111 m/z), (**I**) 1,5- anhydro-D-fructose (163.059 m/z), (**J**) D-phenylalanine (166.085 m/z), (**K**) L-valine (118.086 m/z), (**L**) L-leucine (132.102 m/z), (**M**) dimethyl fumarate (145.049 m/z), (**N**) glutamate (148.060 m/z), (**O**) 2-aminomuconate (158.091 m/z), (**P**) indole-3-propanol phosphate (256.081 m/z), and (**Q**) glutamine (147.077 m/z), across 35 samples.

G200 treatment was significantly separated from G0, C0 and C200 treatments, due to high level of coumarin (258.109 m/z at 2.45 min) (Fig 6A) and azatyrosine (183.085 m/z at 2.54 min) (Fig 6B). G0 treatment showed significantly high level of L-seryl-L-prolyl-L-threonyl-L-seryl-L-seryl-L-alanyl-L-seryl-L-seryl-L-phenylalanine (870.314 m/z at 2.60 min) (Fig 6C), eudistomin I (236.148 m/z at 2.68 min) (Fig 6D), tryptophan (268.102 m/z at 5.40 min) (Fig 6E), nitric acid (190.143 m/z at 2.14 min) (Fig 6F), and chorismatic acid (226.949 m/z at 1.86 min) (Fig 6G) compared to other treatments. On the other hand, C0 and C200 treatments showed significantly high level of difructose anhydride (325.111 m/z at 2.55 min) (Fig 6H), 1,5-anhydro-D-fructose (163.059 m/z at 2.55 min) (Fig 6I), D-phenylalanine (166.085 m/z at 6.40 min) (Fig 6J), L-valine (118.086 m/z at 2.51 min) (Fig 6K), L-leucine (132.102 m/z at 3.77 min) (Fig 6L), dimethyl fumarate (145.049 m/z at 2.54 min) (Fig 6M), and glutamate (148.060 m/z at 2.42 min) (Fig 6N). Although the ion intensity for certain compounds appeared to be different for samples of the same treatment group, PCA analysis showed that all samples clustered to their respective treatment groups (Fig 3). The differences in ion intensity which caused by some differences between samples, which also reflected in the distance of the respective sample to the treatment group in PCA plot (Fig 3), were not significant.

**Fig 7:**
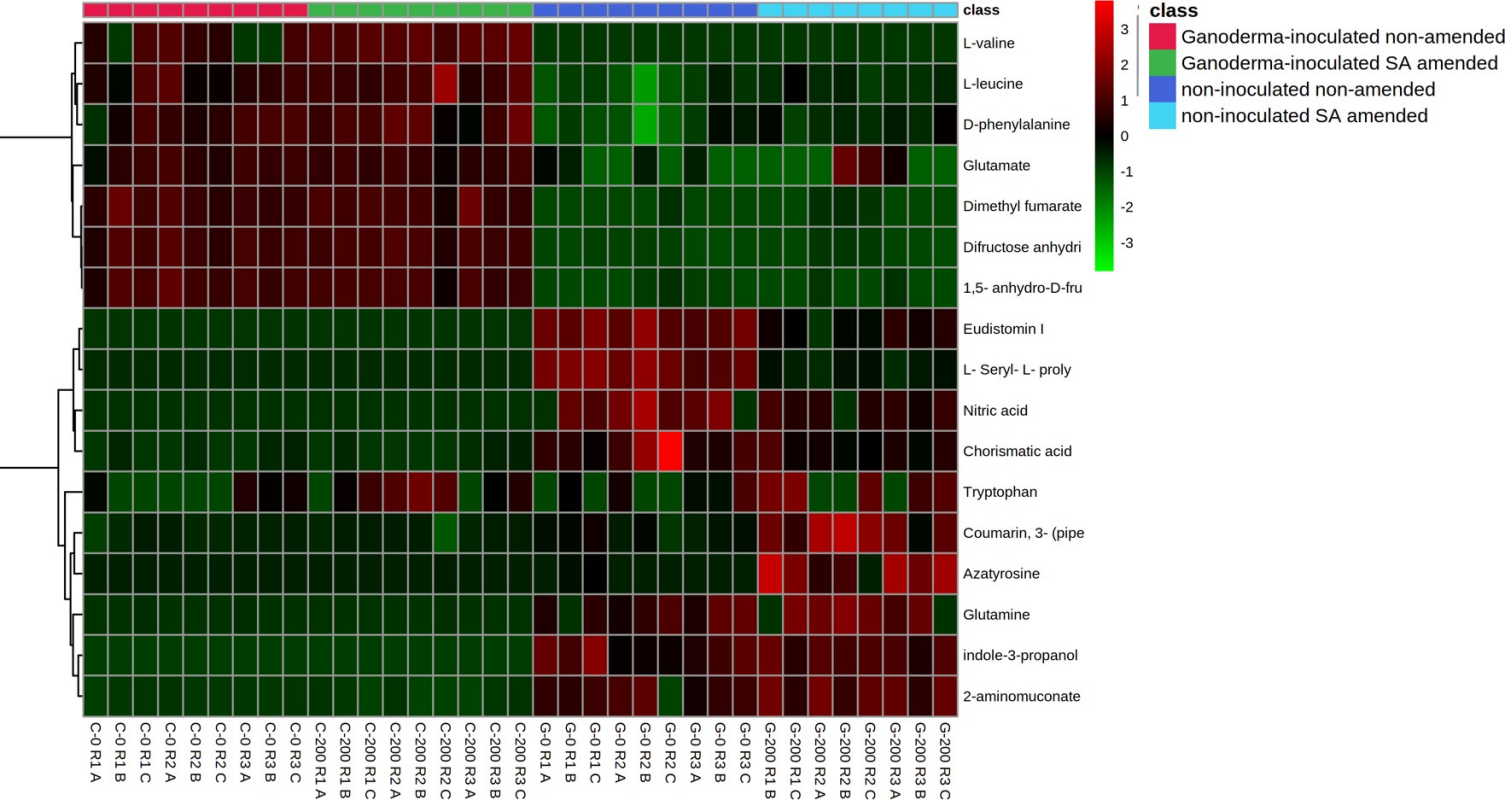
Hierarchically clustered heat map of the metabolites with the highest VIP value with statistical significance. Colors are based on intensity levels and changes in metabolites, where bright red indicates the highest intensities while light green indicates the lowest intensities or complete absence of metabolites.

The heat map of the respective VIP metabolites corresponding to each group was also presented (Fig **7**). The variation in color spectrum is due to the intensity of the compound based on the extracted ion chromatogram (EIC). Rows represent metabolites, and columns represent samples.

## 4. Discussion

### 4.1 Growth response of *G*. *boninense* to salicylic acid

In our experiment, the inhibitory effect of SA on the growth of *G*. *boninense* was significant, which conformed to the findings of Bivi et al. (2012) [11]. The inhibitory effect based on PIRG values increased steadily with increasing concentration of SA (Fig 2). However as shown in Fig 2, our results were three-fold higher as compared to Bivi et al. (2012) [11] for the same isolate of *G*. *boninense* (PER71) and SA at concentration of 200 μg g^-1^ at day 5 after treatment. This contradiction may be due to the addition of small amount of absolute ethanol (0.24% v/v) in preparing the SA solutions, therefore affected the final concentrations of SA in the culture medium.

We also observed regrowth of *G*. *boninense* after complete growth inhibition at the beginning of the experiment, particularly for all isolates treated with 150 and 200 μg g^-1^ SA (Fig 2 and S1–S4 Figs). In other words, the inhibitory effect of SA treatments (150 and 200 μg g^-1^ SA) on the growth of *G*. *boninense* declined over time. In this study, we did not transfer the treated culture onto a fresh medium as commonly performed to check for mycocidal-mycostatic nature of any treatment [51, 52]. Regardless, regrowth was still observed, which implies the ability of *G*. *boninense* to adapt and/or degrade the SA treatment. To date, there has been no known published work on the degradation of SA by *G*. *boninense* to the best of our knowledge. However, Chong (2010) [9] showed that *G*. *boninense* was capable of degrading other phenolic acids, namely syringic acid, caffeic acid, and 4-hydroxybenzoic acid, into non- or less-antifungal compounds. In the experiment, high concentrations of syringic acid (90 and 110 μg mL^-1^) remained stable at the beginning of incubation with *G*. *boninense*, with negligible change. The degradation of syringic acid started at day 10 of incubation and its utilization by *G*. *boninense* reached nearly 100% at day 14 of incubation [9]. High concentrations of SA may cause lysis of hyphae as suggested by Bivi et al. (2012) [11]. Thus, the ability of *G*. *boninense* to degrade SA as a defense response is of great importance to overcome cell lysis and ensure its survivability.

Noticeably, at SA concentration of 100 μg g^-1^ or less, the least aggressive G8 seemed to metabolise SA as a carbon source for its growth (Fig 1 and S1 Fig), similar to the case of *P*. *carotovorum* [16]. In the experiment with *P*. *carotovorum*, growth inhibition was observed at high concentrations, whereas its growth was stimulated in a near-dose dependent manner at low concentrations [16]. *Pectobacterium carotovorum* might have used SA as a carbon source for growth by degrading SA via catechol and gentisate pathways [16, 53]. The observation of high-dose inhibition and low-dose stimulation, also known as hormesis, is common in fungal physiology, under exposure to chemical treatments [54–57]. In a recent study conducted by Surendran et al. (2018) [58], low concentration of SA (1 mM) induced the incidence of *Ganoderma* disease by 60% in their nursery trial, thus reaffirming the low-dose stimulation effect of SA on the growth of *G*. *boninense* as observed in G8 isolate.

### 4.2 Metabolites production by *G*. *boninense* in defence against salicylic acid stress

Formation of reddish zone around the mycelial plug was observed only in SA-amended plates inoculated with *G*. *boninense* (S2–S4 Figs). Since non-inoculated SA-amended plates (C200) did not have the same reddish zone, it was unlikely that the reddish pigmentation arose from the chemical reaction between SA and PDA medium. Therefore, it is plausible that either SA induces *G*. *boninense* to produce metabolite which is reddish in color or the utilization of SA by *G*. *boninense* produces reddish metabolite to account for the reddish pigmentation.

Among the detected compounds from the G200 treatment (Table 1 and Fig 6), only azatyrosine (183.085 m/z) forms reddish compound and hence, the most probable candidate for the reddish pigmentation. Azatyrosine is a precursor of melanin which is produced by *G*. *boninense* as a defense mechanism to protect fungi from environmental stresses such as antimicrobial agents and lytic enzymes [2, 38]. Melanin can be derived from the oxidation of tyrosine or tyrosine-containing protein hydrolysates, by the enzymatic activity of tyrosinase [38]. Moreover, melanin can also be produced from catechol, which is one of the degradation products of SA [38, 59]. SA treatments have been found to induce production of reactive oxygen species (ROS) and cause cell apoptosis in *G*. *lucidum* [60–62]. Similarly, at later stage of infection by *G*. *boninense*, ROS will be produced as a result of lignin degradation, which ultimately may results in formation of phenoxyl radicals [2, 63]. At this stage, fungal mass of *G*. *boninense* often became encrusted and pigmented with melanin. This melanized fungal structure may be important in protecting *G*. *boninense* from the hostile environments [2]. Using the annotated genome sequence of *G*. *boninense*, we confirmed the presence of salicylate hydroxylase gene, which commonly reported to be involved in SA metabolism pathway (i.e., hydroxylation and decarboxylation) to produce catechol [64, 65]. In *G*. *tsugae*, this gene has been hypothesized to have important role in conferring resistance towards SA accumulation during substrate utilization [65]. Thus, it is unsurprising that *G*. *boninense* responded to the SA stress by degrading it, and ultimately hastening the melanin formation.

Furthermore, azatyrosine may alleviate the effect of SA on the growth of *G*. *boninense*. In *G*. *lucidum*, aspirin treatment in which SA is the principal compound reduced fungal biomass, induced apoptosis and increased ganoderic acid production [61, 62]. It was found that the activity of 3’,5’-cyclic adenosine monophosphate (cAMP) signaling was induced in response to the aspirin treatment [61, 62]. Azatyrosine may acts as chemopreventive agent against carcinogenesis by intervening *ras*-mediated signaling pathway, the upstream of cAMP signaling [37]. In basidiomycete, *ras*-signaling influences multiple aspects of morphogenesis such as cell growth, cell differentiation, apoptosis, nuclear distribution in mating interactions, clamp fusion, fruiting body morphology and spore production [66–68]. It is likely that azatyrosine is produced to adapt and/or mitigate the effect of SA treatment, by modulating the developmental stage of *G*. *boninense*.

Coumarin (258.109 m/z) was another metabolite detected at significantly high concentration in G200 treatment (Fig 6A). Coumarin is synthesized via Perkin reaction or *O-*acetylation of salicylaldehyde, while reduction of SA yields salicylaldehyde [69–71]. In a recent study, treatment of SA at 10 mg mL^-1^ enhanced the production of bioactive constituents in fruiting bodies of *G*. *lucidum*, i.e., triterpenoids. Up-regulations of transcripts, namely, *pgm* and *ugp*, which are involved in polysaccharide biosynthesis pathway, contributed to higher production of the triterpenoids [72]. In our study, the SA treatment may have similar stimulating effect on the production of coumarin, which is also present in fruit bodies of several basidiomycetes such as *G*. *lucidum*, *Pleurotus ostreatus* and *Lentinula edodes* [73].

Coumarin is more extensively reported as one of the phenylpropanoid compounds, which is a plant defense compound and is secreted by many plant families as a response to abiotic and biotic stresses [29]. It may act as an antioxidant agent against free radicals and reactive oxidative species (ROS), which are largely produced under stress [30]. The ability of pathogens to secrete plant hormones and hormone analogs to manipulate hormone homeostasis or hormone signaling of host plant has been widely reviewed [74–77]. In most cases, the ability of synthesizing plant hormones or hormone analogs contributes to virulence of the pathogens. Potentially, coumarin is one of many metabolites secreted by *G*. *boninense* to modulate defense-related signaling pathway and/or phyto-hormone homeostasis.

Detection of azatyrosine and coumarin upon SA treatment implicated the role of SA in modulating the developmental switch in *G*. *boninense*. As discussed, both coumarin and azatyrosine are involved in the formation of melanin and/or fruiting bodies of *G*. *boninense*, which takes place at the third stage of infection process [2]. As shown in S2–S4 Figs, SA treatment clearly hastened the formation of melanized mycelia and affected the intensity of the pigmentation for all isolates. Therefore, SA may acts as the cue for the transition in infection stage, directly or indirectly. It is believed that fungi have the ability to sense unfavourable environmental conditions. One of the responses is by triggering the development of fruiting bodies in order to disperse fungal spores for propagation [78–80]. Alternatively, coumarin and azatyrosine may be produced as a result of hypersensitive response (or apoptosis). This contention is supported by the results of aspirin and SA treatments, which induced apoptosis in *G*. *lucidum*, and simultaneously stimulated the production of secondary metabolite, i.e., ganoderic acids [60, 61]. In this study, we also detected low amount of ganoderic acids in both G0 and G200 treatments (S7 Fig). Higher abundance of ganoderic acids were found in G200 treatment compared to G0 treatment (S7 Fig). Similar to *G*. *lucidum*, it is therefore can be postulated that SA treatment can modulate the metabolism of *G*. *boninense*, likely by elevating intracellular ROS, simultaneously or consequently increased its secondary metabolite production [60].

### 4.3 Physiological pathways of *G*. *boninense* to support growth in-vitro

**Fig 8:**
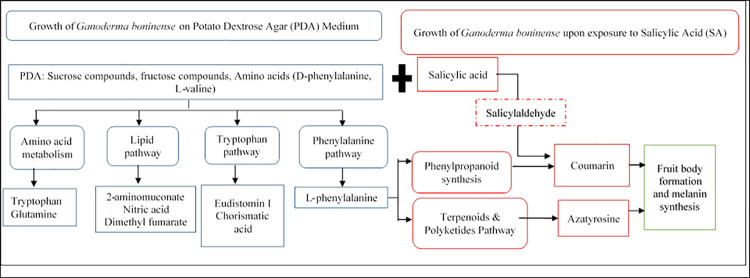
Schematic diagram of *Ganoderma* biosynthetic pathway showing the major pathways in the absence and presence of salicylic acid.

Apart from investigating the metabolomics response of *G*. *boninense* towards SA treatment, the LC-TOF-MS results allowed us to postulate some pathways involved in the growth of *G*. *boninense* on the PDA medium. At least four metabolic pathways were involved in the growth of *G*. *boninense*, as deduced from metabolite compositions in *Ganoderma*-inoculated plates (G0 and G200) compared with their corresponding controls (C0 and C200). *Ganoderma boninense* utilised the nutrients in PDA, i.e., amino acids and sugars, to synthesize more complex compounds, such as aromatic amino acids, oligopeptides, alkaloids and antimicrobial compounds (Table 1) [81]. The pathways include amino acid metabolism, lipid metabolism, tryptophan pathway, and phenylalanine pathway. Fig 8 illustrates the involvement of the metabolites detected in this study in the abovementioned pathways.

A number of metabolites were detected in higher abundance in control groups (C0 and C200) compared to *Ganoderma*-inoculated plates (G0 and G200). These metabolites include: difructose anhydride (325.111 m/z) (Fig 6H), 1,5-anhydro-D-fructose (163.059 m/z) (Fig 6I), D-phenylalanine (166.085 m/z) (Fig 6J), L-valine (118.086 m/z) (Fig 6K), L-leucine (132.102 m/z) (Fig 6L), dimethyl fumarate (145.049 m/z) (Fig 6M), and glutamate (148.060 m/z) (Fig 6N). Based on KEGG database, difructose anhydride and 1,5-anhydro-D-fructose are involved in starch and sucrose metabolism. Dextrose and potato starch in the PDA medium could be the sources of these detected sugars. Noticeably, these sugars were almost absent in the *Ganoderma*-inoculated plates (G0 and G200 treatments). Similarly, D-phenylalanine L-valine, L-leucine, dimethyl fumarate and glutamate, which could be secondary products from amino acid content of potato infusion in the PDA medium, were also depleted in the *Ganoderma*-inoculated plates. Similar to our results, Ahmad et al. (2020) [81] reported the depletion of glucose, tryptophan, tryptamine and aconitate, in the co-culture medium of *G*. *boninense* and *Scytalidium parasiticum*. Together, these imply the utilization of these sugars and nitrogen sources for the fungal growth.

Several metabolites, namely L-valine, and eudistomin I, were shown to have antimicrobial activity against *Mycobacterium* spp., and *Saccharomyces cerevisiae*, respectively (Table 1) [27, 44]. This is not surprising as *Ganoderma* species, particularly *G*. *lucidum*, is well-known for its richness in bioactive components and medicinal values [82]. In a recent study, eudistomin was also detected in co-culture medium of *G*. *boninense* and *S*. *parasiticum* [81]. It is therefore logical to deduce the constitutive secretion of eudistomin by *G*. *boninense*, judging from the negligible antifungal effect of eudistomin on the growth of *G*. *boninense* [81]. During colonisation of ecological niche (e.g, oil palm), the ability to produce antimicrobial compounds may help in competing with other microorganisms [83]. It is extremely relevant to *G*. *boninense*, being a weak saprophytic fungus [84]. These antimicrobial compounds may often benefit the growth and colonisation of *G*. *boninense* in non-axenic environments such as oil palm tissues.

In addition to eudistomin, Ahmad et al. (2020) [81] detected penipanoid A in the co-culture medium. This metabolite was deemed to have minimal antifungal effect against *G*. *boninense*, thus likely to be produced by *G*. *boninense* [81]. Unlike their study, we detected 2-aminomuconate and indole-3-propanol phosphate, to be present only in *Ganoderma*-inoculated plates (G0 and G200) (Fig 6O and 6P). Penipanoid, 2-aminomuconate and indole-3-propanol phosphate, are all share the same intermediate, i.e., anthranilate, in tryptophan metabolism [46, 81, 85]. The presence of antagonistic fungus, i.e., *S*. *parasiticum* may have triggered the expression of silent biosynthetic pathways in *G*. *boninense*, thus secreting new metabolites, i.e., penipanoid A, rather than 2-aminomuconate and indole-3-propanol phosphate as observed in our study [81, 86].

Noticeably, several metabolites were detected at lower concentrations in the presence of SA (G200), compared to non-amended *Ganoderma*-inoculated plate (G0). The metabolites were eudistomin I, L-phenylalanine, nitric acid and chorismatic acid. The presence of SA, which may act either as stress factor, or carbon source, may have altered the metabolic pathways of *G*. *boninense*. This implies the metabolic versatility of *G*. *boninense* in responding to the surrounding stimuli, which is important in fungal physiology and pathogenicity, for nutrient assimilation and adaptation to host-imposed stress [87]. The absence of L-phenylalanine in the G200 treatment provided concrete evidence for this metabolic versatility. L-phenylalanine is an intermediate metabolite to produce coumarin via phenylpropanoid biosynthesis pathway [34]. It is likely that *G*. *boninense* utilized this phenylalanine to produce coumarin in the presence of SA treatment. Earlier findings on the ability of *G*. *boninense* to catabolize a range of phenols, namely syringic acid, caffeic acid, 4-hydroxybenzoic acid, and catechin, have also hinted the metabolic versatility of *G*. *boninense* [9, 81]. On that account, *G*. *boninense* is undeniably a fungal pathogen that is metabolically versatile, which allows it to mitigate various environmental stresses for survival.

## 5. Conclusion

This study provides the first evidence detailing the metabolic responses of *G*. *boninense* that enable consequent modulation and switching of its developmental growth stage to combat salicylic acid stress. The production of coumarin and azatyrosine, which are the pre-cursors of melanin and/or fruit bodies, probably reduces the negative impacts of salicylic acid over time. In the absence of salicylic acid, *G*. *boninense* utilizes carbohydrate and amino acid for its growth and undergoes at least four metabolic pathways, namely amino acid metabolism, lipid pathway, tryptophan pathway and phenylalanine pathway. The capability to produce antimicrobial metabolites may benefit the survival of *G*. *boninense* during colonisation of oil palm tissues through successfully competing with other microorganisms. At lower concentrations of the treatment, the least aggressive *G*. *boninense* isolate could utilize salicylic acid as a carbon source to promote its growth. Together, these findings imply the pronounced metabolic versatility of *G*. *boninense* in mitigating changes to its growing environment, which is by switching its developmental stage as illustrated in this study.

## Supporting information

S1 Fig Average radial growth of three *G*. *boninense* isolates treated with different concentrations of SA, at Day 5, 7, 9, and 12 after treatment [adapted from Ong et al. 2018 [88]]. (TIF)

S2 Fig *Ganoderma boninense* PER71 isolate (moderately aggressive) cultured on non-amended medium (0 μg g^-1^), and SA-amended (100 and 200 μg g^-1^) media, at Day 5, 9, 14, and 21 days after treatment. (TIF)

S3 Fig *Ganoderma boninense* G8 isolate (least aggressive) cultured on non-amended medium (0 μg g^-1^), and SA-amended (100 and 200 μg g^-1^) media, at Day 5, 9, 14, and 21 days after treatment. (TIF)

S4 Fig *Ganoderma boninense* G10 isolate (most aggressive) cultured on non-amended medium (0 μg g^-1^), and SA-amended (100 and 200 μg g^-1^) media, at Day 5, 9, 14, and 21 days after treatment. (TIF)

S5 Fig T2 range analysis for outlier determination. (TIF)

S6 Fig DmodX analysis to determine distance to model plots in X space. (TIF)

S7 Fig Detection of ganoderic acids in each treatment group. (TIF)
