# Supplementary figures and images for: Growth modulation and metabolic responses of Ganoderma boninense to salicylic acid stress
Source: PLoS One. 2021 Dec 31;16(12):e0262029. doi: 10.1371/journal.pone.0262029 (PMC8719765; doi:10.1371/journal.pone.0262029)

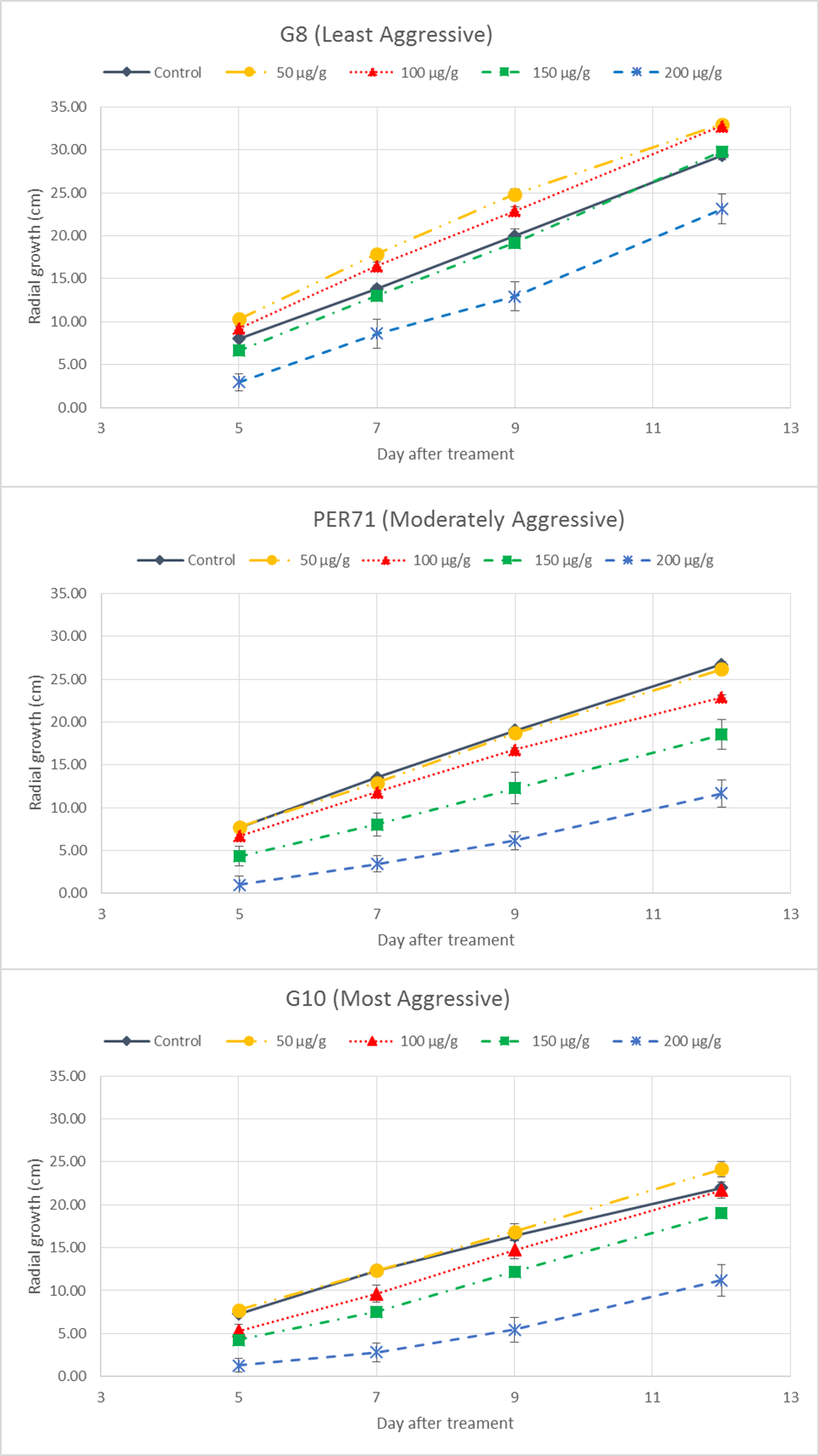

Supplement: S1 Fig — (TIF) [file pone.0262029.s001.tif]

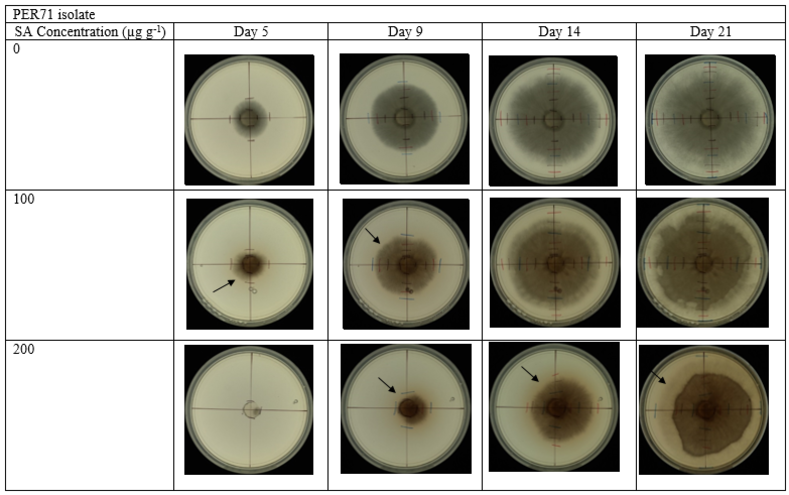

Supplement: S2 Fig — (TIF) [file pone.0262029.s002.tif]

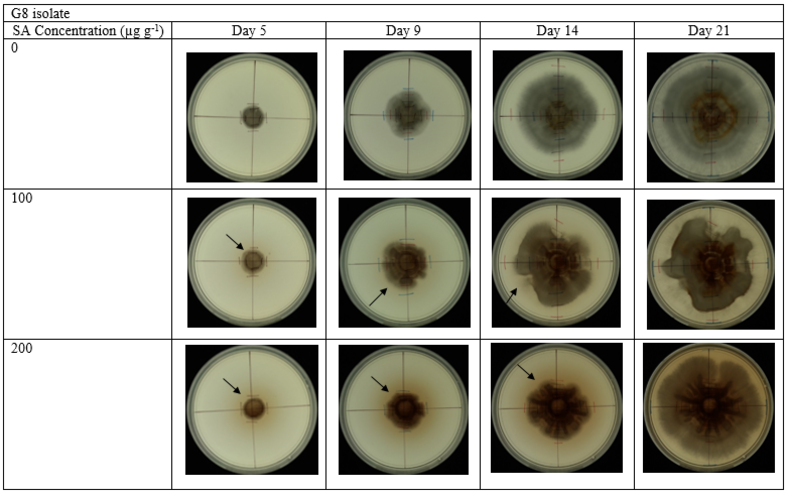

Supplement: S3 Fig — (TIF) [file pone.0262029.s003.tif]

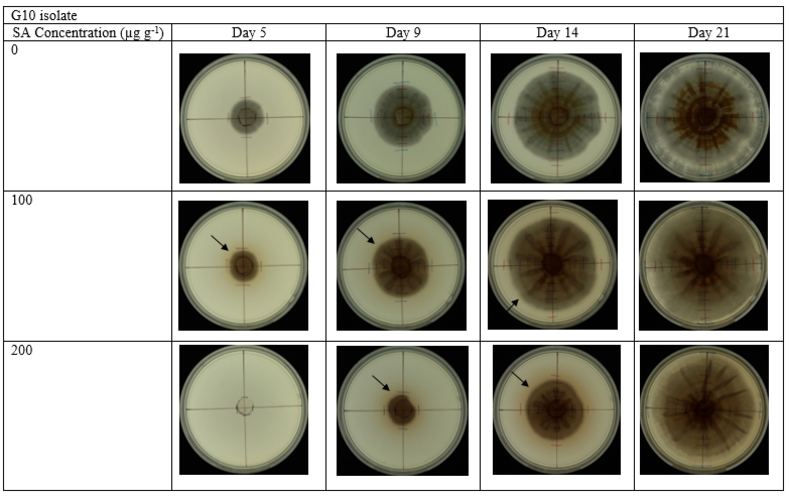

Supplement: S4 Fig — (TIF) [file pone.0262029.s004.tif]

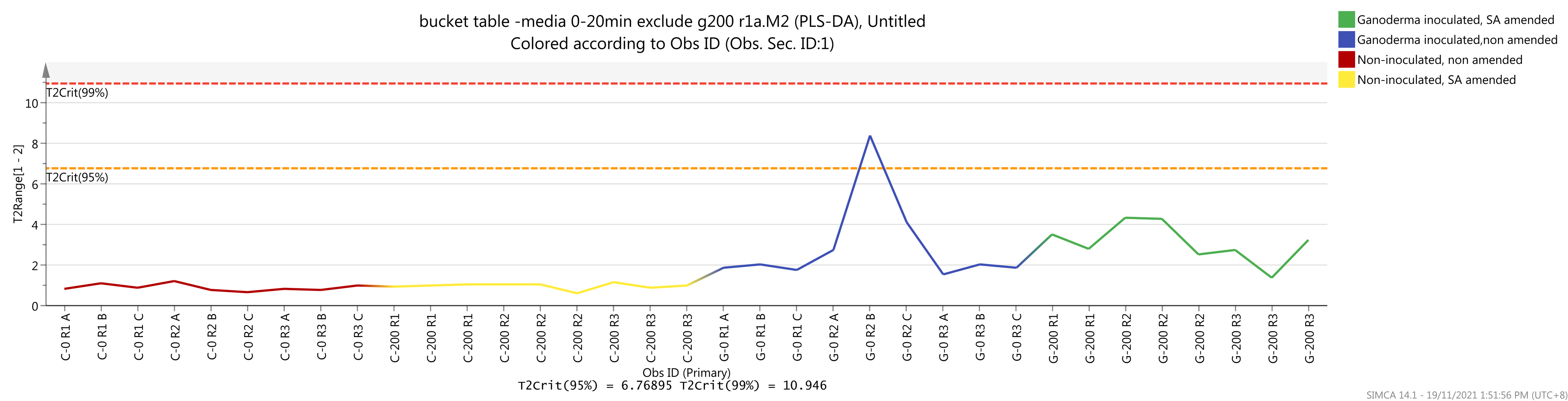

Supplement: S5 Fig — (TIF) [file pone.0262029.s005.tif]

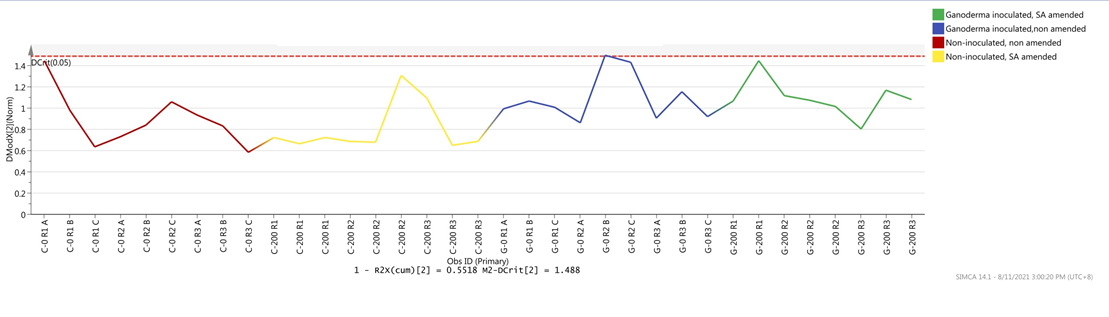

Supplement: S6 Fig — (TIF) [file pone.0262029.s006.tif]

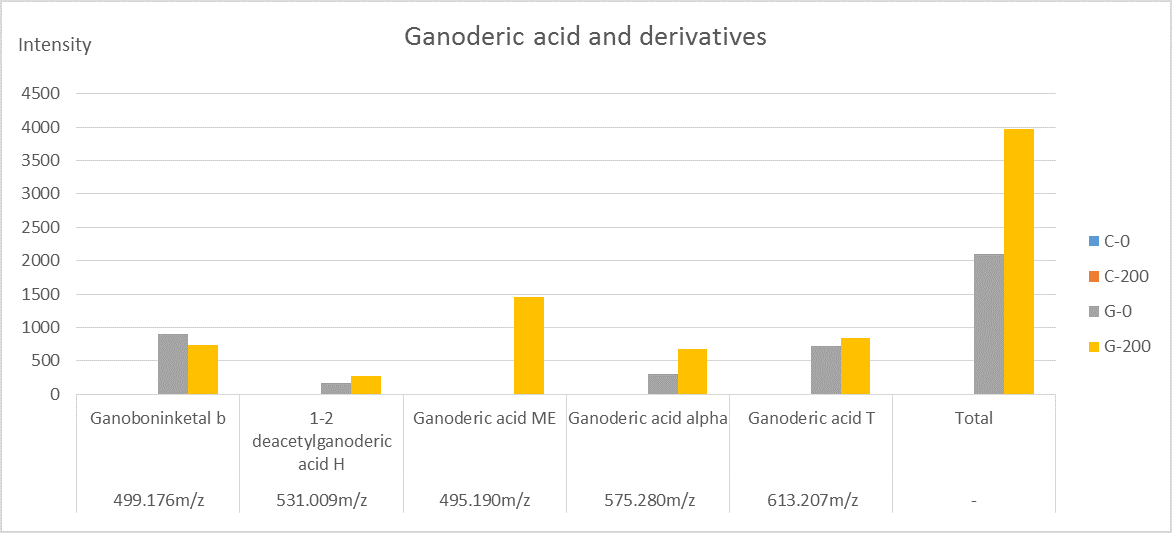

Supplement: S7 Fig — (TIF) [file pone.0262029.s007.tif]
